# Does emergency department use and post-visit physician care cluster geographically and temporally for adolescents who self-harm? A population-based 9-year retrospective cohort study from Alberta, Canada

**DOI:** 10.1186/s12888-016-0941-3

**Published:** 2016-07-11

**Authors:** Rhonda J. Rosychuk, David W. Johnson, Liana Urichuk, Kathryn Dong, Amanda S. Newton

**Affiliations:** Department of Pediatrics, University of Alberta, Edmonton, AB Canada; Women & Children’s Health Research Institute, Edmonton, AB Canada; Department of Pedatrics, Cumming School of Medicine, University of Calgary, Calgary, AB Canada; Department of Pharmacology and Physiology, Cumming School of Medicine, University of Calgary, Calgary, AB Canada; Addiction & Mental Health, Alberta Health Services, Edmonton, AB Canada; Department of Psychiatry, University of Alberta, Edmonton, AB Canada; Department of Emergency Medicine, University of Alberta, Edmonton, AB Canada; Department of Pediatrics, University of Alberta, Rm 3–524, Edmonton Clinic Health Academy (ECHA), 11405 87 Avenue NW, Edmonton, AB T6G 1C9 Canada

**Keywords:** Adolescence, Space-time clustering, Disease clustering, Emergency services, Self-harming behavior

## Abstract

**Background:**

Clustering of adolescent self-harming behaviours in the context of health care utilization has not been studied. We identified geographic areas with higher numbers of adolescents who (1) presented to an emergency department (ED) for self-harm, and (2) were without a physician follow-up visit for mental health within 14 days post-ED visit.

**Methods:**

We extracted a population-based cohort of adolescents aged 15–17 years (*n* = 3,927) with ED visits during 2002–2011 in Alberta, Canada. We defined the case as an individual with one or more ED presentations for self-harm in the fiscal year of the analysis. Crude case rates were calculated and clusters were identified using a spatial scan.

**Results:**

The rates decreased over time for ED visits for self-harm (differences: girls −199.6/100,000; *p* < 0.01; boys −58.8/100,000; *p* < 0.01), and for adolescents without a follow-up visit within 14 days following an ED visit for self-harm (differences: girls −108.3/100,000; *p* < 0.01; boys −61.9/100,000; *p* < 0.01). Two space-time clusters were identified: (1) a North zone cluster during 2002–2006 (*p* < 0.01) and (2) a South zone cluster during 2003–2007 (*p* < 0.01). These clusters had higher numbers of adolescents who presented to the ED for self-harm (relative risks [RRs]: 1.58 for cluster 1, 3.54 for cluster 2) and were without a 14-day physician follow-up (RRs: 1.78 for cluster 1, 4.17 for cluster 2). In 2010/2011, clusters in the North, Edmonton, and Central zones were identified for adolescents with and without a follow-up visit within 14 days following an ED visit for self-harm (*p* < 0.01).

**Conclusions:**

The rates for ED visits for adolescents who self-harm and rates of adolescents without a 14-day physician follow-up visit following emergency care for self-harm decreased during the study period. The space-time clusters identified the areas and years where visits to the ED by adolescents for self-harm were statistically higher than expected. These clusters can be used to identify locations where adolescents are potentially not receiving follow-up and the mental health support needed after emergency-based care. The 2010/2011 geographic cluster suggests that the northern part of the province still has elevated numbers of adolescents visiting the ED for self-harm. Prospective research is needed to determine outcomes associated with adolescents who receive physician follow-up following ED-based care for self-harm compared to those who do not.

## Background

In Canada, several recently publicized clusters of deaths by suicide among young people [1, 2] have focused attention on ‘point clusters’—unusually high numbers of suicides occurring in a close geographic location and brief time period. Such clusters have also been documented globally for individuals of all ages, [3–7] although clustering has been found to be up to four times more common among adolescents and young adults than among other age groups [3]. The study of self-harm clusters—clusters of non-fatal self-poisoning or self-inflicted harm irrespective of suicidal intent—is uncommon even though the average lifetime prevalence of self-harming behavior is much higher among adolescents (ranging from 17–39 % in adolescence) than an outcome of death by suicide (9.0 per 100,000 for adolescents aged 15 to 19 years) [8–10]. While deliberate self-harm often occurs in the absence of suicidal intent, it is considered a clear sign of emotional distress that may result in accidental death or serious injury. Compared to other age groups, young people are more likely to report suicidal thoughts and self-harm, elevating their risk for a suicide attempt or death by suicide at a later date [11]. Non-suicidal self-harm has also been shown to be predictive of future suicide attempts among adolescents with treatment-resistant depression [12]. Further, in a recent study, Swanson and Colman found that exposure to suicide is associated with increased suicidal ideation and attempt, [13] results that highlighted self-harm risks are spatially and temporally bound.

The study of self-inflicted harm clusters in the context of health care seeking has yet to be conducted, but presents an opportunity to identify important spatio-temporal trends in care, particularly among young people who may be at greater risk for negative health and psychological outcomes. We focused this study on spatial-temporal trends in emergency department (ED) visits made by adolescents who self-harm and post-ED follow-up visits made to physicians. Among young people who self-harm, approximately one in eight will present to an ED for related care [14–18]. These individuals are considered to be at higher risk for subsequent mortality compared to those who do not present to the ED for care [19] and have a higher prevalence of mental disorders among them [14]. Thus, for these young people, the period immediately following an ED visit is an important time for risk reduction and psychiatric stabilization [20–22].

Recent large-scale studies indicate that 44–75 % of ED visits made by young people for self-harm result in discharge home [17–19, 23–25]. This disposition is considered appropriate for those who are not actively suicidal, do not have access to lethal means, and have a responsible adult to ensure their safety [26]. For these young people, referral to urgent outpatient mental health care may be recommended as follow-up to the ED visit [20]. This recommendation is based on known vulnerabilities of this population, [14, 16] and clinical acumen that follow-up mental health care can promote and sustain the child’s safety, address psychosocial support needed by the child and family in the post-crisis period, and further explore mental health and coping needs of the child and family. Several studies have demonstrated, however, that receipt of follow-up services does not occur for the majority of young people, [25, 27] and that ED visit rates for self-harm and post-ED follow-up visit rates after such visits vary geographically. In accordance, in this study, we extracted population-based data to examine emergency mental health care and follow-up care for adolescents (age 15–17 years) in Alberta, Canada. We describe the adolescents who (1) presented to the ED for self-harm, and (2) presented to the ED for self-harm but did not have a mental health-related physician follow-up visit within 14 days after an ED visit. Using a statistical surveillance technique, we identified geographic areas with higher numbers than expected of adolescents defined by (1) and (2).

## Methods

### Data sources and variable description

Alberta Health provided the population-based data from two databases: (1) the Ambulatory Care Classification System (ACCS) database which records ambulatory care visits to all Alberta government funded facilities (including 104 EDs) and (2) the Alberta Health Care Insurance Plan cumulative population registry which contains demographic and population data. The ACCS database has a main diagnosis field and nine additional fields to capture diagnosis data (Canadian Enhancement of International Classification of Diseases, 10th Revision; ICD-10-CA). Among all ED visits, most of the visits were made by adolescents aged 15 to 17 years. Thus, all ED visits made by 15 to 17 year olds in Alberta between April 1, 2002, and March 31, 2011, where any diagnosis field had a diagnostic code for self-harm (X60-X84, T71), were extracted. While self-harm intent (i.e., behaviour with suicidal vs. non-suicidal intent) is assessed by ED clinicians, this intent is not captured in the ACCS database hence Silverman et al.’s classification of ‘undetermined suicide-related behaviour’ applies to our study [28].

Geographic data were geo-coded to 70 sub-regional health authorities (sRHAs) that constitute five provincial health zones (North, Edmonton, Central, Calgary, South). The 70 sRHAs have diverse population sizes (ranging from 367 to 5,390 in 2011) and the large geographic areas in the north are sparsely populated. Latitudes and longitudes for population-based geographic centres (centroids) were provided by Alberta Health. Population data included counts by sex, age in years, and sRHA of residence at fiscal year end.

Adolescents with ED visits were linked with the Physician Claims File to obtain all physician claims (hereafter follow-up visits) within 14 days of the ED visit. This linkage provided the date of the physician claim and up to three diagnosis fields (International Classification of Diseases, 9th Revision – Clinical Modification; ICD-9-CM). We identified mental health physician follow-up visits (those claims with either the first diagnosis field code as 291.x-292.x, 295.x-298.x, 300.x-309.x, 311, 312.x-314.x, 980.x, 981, 982.x, 986, 987.x, 994.7, E95.x, or any additional diagnostic fields that matched the intentional self-harm category code E95.x, 994.7). We used these data to identify all adolescents who had at least one ED visit for self-harm but did not have any mental health-related physician follow-up visits within 14 days of the ED visit. Although follow-up care is recommended following ED visits for intentional self-harm, no specific timeframe is recommended. Many publicly funded emergency/crisis health care services within Alberta have teams with internal mandates to follow up within 48 h to 7 days after discharge, depending on the nature/risk of the presenting concern. Given the discussion in the published literature [17, 19–22, 26] and recommendations from an international consensus group, [29] which proposed standardized measures for follow-up care between 7 days and 30 days after discharge from hospital, we felt a follow up time frame of 14 days reflected an important window of follow up care after the index event, even among those adolescents considered to be at lower risk for further harm after ED evaluation.

### Statistical analysis

Numerical summaries (e.g., counts, percentages) were used to describe the demographic characteristics of the adolescents presenting to the ED for deliberate self-harm. Crude rates, and corresponding 95 % confidence intervals (CIs), were calculated for each sex and sex by time interactions were assessed through multiple linear regression. ED visits were excluded from analyses if sRHA of residence was missing. Data were analyzed using S-Plus software [30].

The Kulldorff-Nagarwalla (KN) spatial scan test was used to identify geographic areas with excess numbers (clusters) of adolescents with ED visits for self-harm ED and adolescents without a 14-day physician follow-up after the index ED visit. The KN spatial scan is a popular method for identifying clusters and has been used previously to identify clusters in mental health diagnoses (e.g., depression [31] and self-inflicted harm [4, 6, 32–34]). The software SaTScan [35] was used to implement the KN spatial scan test. In each scan, a cylindrical window with a circular geographical area as the base and a time period as the height moves across the study area and period. We used a space window of up to 50 % of Alberta’s population and a time window of 1 year. All tests used the sRHAs as the geographic boundaries and were adjusted by sex. Further, we used the KN test to identify sRHAs that were statistically significant over time (spatio-temporal clustering). A *p*-value (*p*) less than 0.05 was considered to be statistically significant. The Manifold System [36] was used to produce maps of results.

We will followed the STROBE guidelines (Strengthening the Reporting of Observational Studies in Epidemiology) [37] for the reporting of observational studies.

## Results

### Study cohort description

**Fig. 1:**
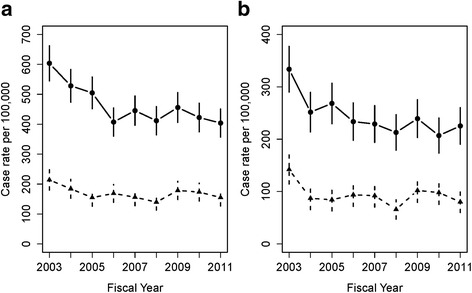
Crude case rates per 100,000 adolescents aged 15–17 years by sex over time. **a** adolescents with ED visits for deliberate self-harm; (**b**) adolescents with an ED visit for self-harm but without a 14-day physician follow-up. females (○) and males (∆)

During the study period, 3,927 adolescents aged 15–17 years made 4,453 ED visits for self-harming behavior. The most common self-harm method was self-poisoning (65.5 %), followed by self-cutting (27.4 %). Asphyxiation was the least commonly reported (2.2 %). The majority of the ED visits occurred in the major urban areas of Edmonton and Calgary (Table 1). Females had disproportionately more ED visits (72.2 %) than would be suggested by the population distribution (48.5 %, Fig. 1a, *p* < 0.01). Approximately 55 % of adolescents with an ED visit for self-harm did not have a mental health follow-up with a physician within 14 days (Table 1). Females had fewer follow-up visits to physicians (Fig. 1b). Rates of ED visits for self-harm decreased from 2002 to 2011 for girls (from 603.5 to 403.8 per 100,000 adolescents, *p* < 0.01) and boys (from 213.9 to 155.0 per 100,000 adolescents, *p* < 0.01). Among the adolescents with ED visits for self-harm, the rates of those without a 14-day physician follow-up decreased over time for girls (from 333.6 to 225.3 per 100,000 adolescents, *p* < 0.01) and boys (from 142.1 to 80.2 per 100,000 adolescents, *p* < 0.01). There was a statistically significant sex by time interaction for ED visits for self-harm (*p* = 0.030) but not for the 14-day physician follow-up outcome (*p* = 0.107).

**Table 1:** Patient sociodemographic and geographic characteristics

	Adolescents aged 15–17 years with an ED visit		Adolescents aged 15–17 years without a 14-day physician follow-up visit		Alberta population aged 15–17 years
	Total	Fiscal Years	Total	Fiscal Years	
	n (%)	Median (Range)	n (%)	Median (Range)	n (%)
All	3,927	418 (384 to 543)	2,153 (54.8)	233 (200 to 326)	145,389
Sex					
Female	2,834 (72.2)	305 (275 to 394)	1,533 (71.2)	164 (146 to 225)	70,571 (48.5)
Male	1,093 (27.8)	122 (99 to 149)	620 (28.8)	69 (49 to 101)	74,818 (51.5)
Zone					
North	719 (18.3)	77 (67 to 94)	429 (19.9)	47 (36 to 58)	19,559 (13.5)
Edmonton	1,395 (35.5)	147 (124 to 218)	798 (37.1)	86 (66 to 140)	43,947 (30.2)
Central	564 (14.4)	60 (43 to 88)	319 (14.8)	33 (23 to 58)	18,865 (13.0)
Calgary	926 (23.6)	100 (85 to 130)	429 (19.9)	47 (32 to 64)	51,169 (35.2)
South	323 (8.2)	35 (26 to 42)	178 (8.3)	20 (11 to 25)	11,849 (8.1)

### Geographical and temporal clustering

**Fig. 2:**
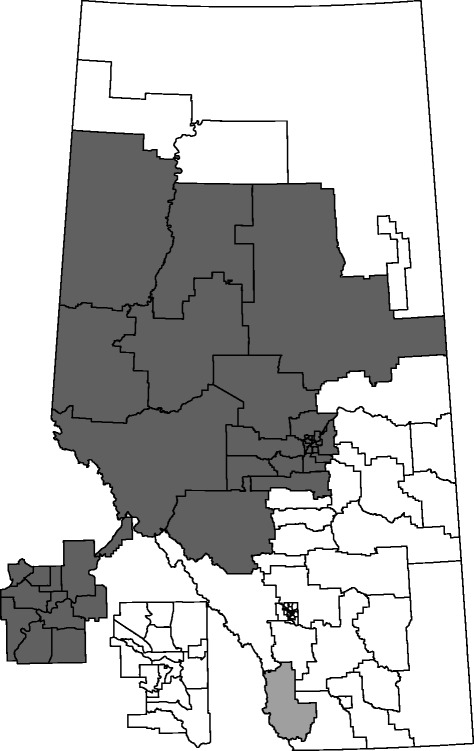
Clusters identified over space and time. For adolescents aged 15–17 years with ED visits for self-harm and without a 14-day physician follow-up after an ED visit. Cluster 1 in dark grey; Cluster 2 in light grey

Two potential spatio-temporal clusters of adolescents with ED visits for self-harming behavior were identified by the KN method when examining the whole study period. The annual number of cases per 100,000 was 304.5 for Alberta. The first potential cluster was concentrated in the north-western area of Alberta during April 1, 2002, to March 31, 2006 (435.7 annual cases per 100,000, *p* < 0.001). A single sRHA in the southwest was identified as a second potential cluster during April 1, 2003, to March 31, 2007 (1,073.9 annual cases per 100,000, *p* < 0.001). These two clusters had relative risks of 1.58 and 3.54, respectively (Table 2, Fig. 2). These same sRHAs were identified as clusters of adolescents without 14-day follow-up and had relative risks of 1.78 and 4.17, respectively.

**Table 2:** Clusters identified over space and time

Cluster	Time Frame	Location (sRHA)	Population		Cases	Expected Cases	Observed/Expected	Relative Risk	*p*-value
1	April 2002 to March 2006	27 Clearwater	59,652	(a)	1,027	717.84	1.43	1.58	<0.001
		28 Brazeau		(b)	612	393.55	1.56	1.78	<0.001
		29 Wetaskiwin-Hobbema							
		41 St. Albert							
		42 Edmonton Castle Downs							
		43 Edmonton Woodcroft							
		44 Edmonton Eastwood							
		45 Edmonton North Central							
		46 Edmonton North East							
		47 Edmonton Bonnie Doon							
		48 Edmonton West Jasper Place							
		49 Edmonton Twin Brooks							
		50 Edmonton Mill Woods							
		51 Sherwood Park							
		52 Strathcona County							
		53 Thorsby							
		54 Leduc Office							
		55 Beaumont							
		56 Westview							
		57 Sturgeon County							
		58 Fort Saskatchewan							
		59 Aspen West							
		60 Aspen Central							
		61 Aspen North							
		63 Peace NW							
		64 Peace NE							
		65 Peace SE							
		66 Peace SW							
2	April 2003 to March 2007	1 Crowsnest Pincher Creek	620	(a)	28	7.94	3.53	3.54	<0.001
				(b)	18	4.35	4.14	4.17	0.006

*sRHA* sub-Regional Health Authority

Legend: (a) adolescents with ED visits for self-harm; (b) adolescents without a 14-day physician follow-up after an ED visit for self-harm

**Fig. 3:**
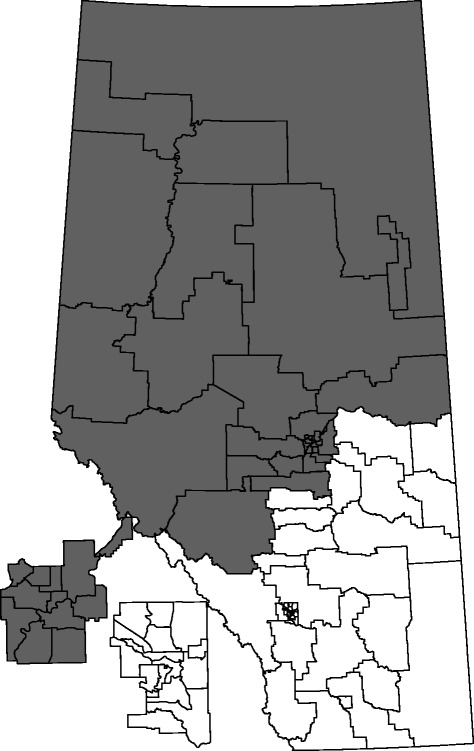
Cluster identified over space during 2010/2011. For both adolescents aged 15–17 years with ED visits for self-harm and without a 14-day physician follow-up after an ED visit. Cluster in dark grey

To identify purely geographical clusters, we applied the KN test to data from each year separately and focused our results on the last two fiscal years. In 2009/2010, one potential cluster of adolescents with ED visits for self-harm was identified that contained seven of the sRHAs from the North zone (*p* < 0.001). The relative risk for this potential cluster was 2.71. The same sRHAs also formed a cluster of adolescents without follow-up (*p* < 0.001) and had a relative risk of 3.75. In 2010/2011, one potential cluster of adolescents with ED visits for self-harm was identified that contained all the sRHAs in the North and Edmonton zones (Fig. 3), as well as three north-western sRHAs in the Central zone (*p* < 0.001). The relative risk for this potential cluster was 1.66. The same cluster was also the only potential cluster identified for the adolescents without physician follow-up (*p* = 0.001) within 14 days. The relative risk for this potential cluster was 1.85.

## Discussion

Recent Canadian and United States investigations of time trends in ED visits for intentional self-harm among adolescents have documented changes. In Ontario during 2002 to 2011, incident rates decreased between 2002/03 to 2006/07, but not thereafter, for adolescents aged 12 to 17 years [38]. In the United States, rates per 1,000 increased from 2.57 to 4.53 during 1993 to 2008 for adolescents aged 15–19 [39] while for those aged 10 to 18 years a National Trauma Data Bank study showed ED visits for self-harm increased from 2009 to 2012 [40]. Our nine-year Canadian population-based study showed the overall rate of ED visits by sex declined during the study period. The observed sex by time interaction for ED visits for self-harm may reflect the gender paradox noted by others in the literature; although not fully understood, it may be that girls seek more opportunities for intervention over time [41, 42]. The observed sex differences in rates of those adolescents without a 14-day physician follow-up (rates 2011, girls versus boys: 225.3 versus 80.2 per 100,000 adolescents) may also reflect the gender paradox. As this study was not designed to investigate reasons for the sex by time interaction or sex differences in health care utilization, further study is warranted and may contribute to understanding the gender paradox observed among young people who deliberately self-harm.

A novel contribution of our study to the literature is our use of a spatial scan to identify geographical-temporal clusters that had higher numbers of adolescents who presented to the ED for self-harm than expected by chance. These clusters are different than the ‘point clusters’ of suicide in that the lack of disaggregated data does not permit specific locations to be used and the focus was on self-harm rather than suicide. Nonetheless, this identification of clusters does provide insights in to the time frame and geographic locations where statistically higher numbers of adolescents have presented to EDs for self-harm. Follow-up to this body of literature is now necessary to determine if rates reflect clinically important changes to health care utilization (e.g., changing access to other services; diversion of adolescents who self-harm to other types of settings such as community supports, employee and family assistance programs, self-help supports), changes to health insurance status (e.g., Affordable Health Care for America Act), and/or whether the type or severity of self-harm is changing and reflected though ED visits.

A unique and important contribution from our study was the identification of geographic and temporal variations in the number of adolescents who were without 14-day physician follow-up after an ED visit for self-harm. Although follow-up mental health care following the ED visit would be recommended and an expected positive outcome designed to promote further risk reduction and psychiatric stabilization, [26] of the nearly 4,000 adolescents with ED visits for self-harm in our study, about 55 % did not have a physician follow-up visit for mental health care after the ED visit. This result is important to note because there are benchmarks for post-ED follow-up care, including increasing the rates of follow-up and coordination between health care service points, as part of the national strategy for suicide prevention in Canada [43]. We identified two potential geographical-temporal clusters where follow-up was absent and, as well, some potential geographic clusters during the 2009/2010 and 2010/2011 fiscal years. The potential clusters were the same for the analyses of adolescents with ED visits for self-harm and those with ED visits but without physician follow-up mental health care. These identified clusters were mainly in the north and central areas of Alberta and may be real or spurious. The sRHAs represented by the clusters included both urban and rural populations and also contain the majority of the First Nations Communities within Alberta. These findings could indicate that youth in the Northern or Central parts of the province (that are more rural) may be more vulnerable to mental health problems leading to self-harm (e.g., anxiety, mood disorders) than youth in other parts of the province. Other information would be required to verify if this is the case and to determine potential factors for the higher ED visit rates in these clusters (e.g., time of presentation – which would impact access to services; social and economic disparities such as unemployment, low income, housing difficulties; access and transportation issues; [44] number of prior self-harm events; First Nation status). Based on these results, prospective research is needed to determine outcomes associated with adolescents who receive physician follow-up following ED-based care for self-harm compared to those who do not. Investigation of the role and impact of follow-up with non-physician community supports should also be considered for future studies. This aspect is important because availability of community mental health non-physician supports is often far greater than physician services.

Although not central to our study, similar to other studies of emergency health care use for self-harm among adolescents, more ED visits were made by females for self-harming behavior [24, 38]. These consistent findings suggest that school-based mental health prevention efforts should address self-harming behavior among young females by discussing when and how to seek help and who they could talk to about their behavior.

We acknowledge that our study has several limitations. First, like all cluster detection methods, the spatial scan cannot determine if an identified cluster is clinically important or not. For example, a clinically important cluster could be due to higher severity of self-harm in adolescents requiring emergency care or an area with less availability of other health services. In contrast a spurious cluster can result from, for example, variation in coding practices. This limitation is true for all cluster detection analyses and further targeted epidemiological research is needed to determine if a detected cluster is clinically relevant. Second, although the KN spatial scan works well for identifying circular and primary clusters that are close in proximity, it can miss secondary or irregularly shaped clusters. Third, our case definitions of an adolescent with at least one ED visit for self-harm during the study period or an adolescent without follow-up do not include all adolescents who self-harm. For example, the definitions exclude any adolescents who self-harm that seek health services outside the ED. Further, it is not possible to distinguish between suicidal and non-suicidal acts when self-harming behaviors are identified by ICD diagnostic codes. Fourth, our study includes several other potential limitations: (1) we have assumed that the sRHA of residence has not changed over time, (2) the time of the ED presentation could impact which services were available in the ED to assist with discharge planning, and (3) only data for physician follow-ups were available. The latter is important to note, especially in large geographic rural areas of Alberta (such as Northern Alberta), because there is a general shortage of psychiatrists in these areas. A large proportion of the psychiatrists serving these areas is based in urban centres and provides travelling clinics to rural sites. Accordingly, the majority of mental health follow-up services for residents in those areas may be with general physicians (e.g., a family physician) or with non-physicians (e.g., private counselling, community mental health clinics, employee and family assistance programs, other community services, self-help groups or online services). Unfortunately, data for non-physician mental health professional services are not captured in the databases and such data are not available for analysis. Finally, we have restricted our study to 15 to 17 year olds and our conclusions would be limited to this age group. Notwithstanding these limitations, our study is based on a long study period with large, population-based databases that are comprehensive and complete with respect to physician follow-up data.

## Conclusion

Using large, population-based databases over nine fiscal years, we identified geographic and temporal variations in the numbers of adolescents aged 15–17 presenting to EDs for self-harm and the number of adolescents presenting to EDs without 14-day physician follow-up for mental health care. The potential clusters identified may represent geographic areas with higher harm severity or a lower availability of non-ED health services. The clusters are not all likely to have occurred by chance. Further investigation and intervention is warranted to document and address the unmet needs of adolescents presenting to the ED with self-harm.

## Abbreviations

ED, emergency department; ACCS, ambulatory care classification system; RHA, sub-regional health authority; CIs, confidence intervals
